# Novel Gene Biomarkers Specific to Human Mesenchymal Stem Cells Isolated from Bone Marrow

**DOI:** 10.3390/ijms252211906

**Published:** 2024-11-06

**Authors:** Sandra Muntión, Elena Sánchez-Luis, María Díez-Campelo, Juan F. Blanco, Fermín Sánchez-Guijo, Javier De Las Rivas

**Affiliations:** 1 Cell Therapy Area, Department of Hematology, Institute of Biomedical Research of Salamanca-Hospital Universitario de Salamanca (IBSAL-HUS), 37007 Salamanca, Spain; smuntion@usal.es (S.M.); ferminsg@usal.es (F.S.-G.); 2 Centro en Red de Medicina Regenerativa y Terapia Celular de Castilla y León, Institute of Biomedical Research of Salamanca-Hospital Universitario de Salamanca (IBSAL-HUS), 37007 Salamanca, Spain; 3 Bioinformatics and Functional Genomics Group, Cancer Research Center (CiC-IBMCC), Consejo Superior de Investigaciones Científicas (CSIC) and University of Salamanca (USAL), 37007 Salamanca, Spain; elenasl@usal.es; 4 Bioinformatics Functional Genomics CANC-14 Group, Institute of Biomedical Research of Salamanca (IBSAL), 37007 Salamanca, Spain; 5 Department of Hematology, Center for Biomedical Research in Network of Cancer (CIBERONC), Institute of Biomedical Research of Salamanca-Hospital Universitario de Salamanca (IBSAL-HUS), 37007 Salamanca, Spain; mdiezcampelo@usal.es; 6 Department of Medicine, Faculty of Medicine, University of Salamanca (USAL), 37007 Salamanca, Spain; jfblanco@usal.es; 7 Department of Trauma and Orthopedic Surgery, University Hospital of Salamanca (IBSAL-HUS), 37007 Salamanca, Spain

**Keywords:** cell marker, transcriptomic, stem cell, Mesenchymal cell, MSC, fibroblast, gene expression, CD marker, hematopoietic cell, bioinformatics

## Abstract

In this paper, we present a comparative analysis of the transcriptomic profile of three different human cell types: hematopoietic stem cells (HSCs), bone marrow-derived mesenchymal stem cells (MSCs) and fibroblasts (FIBs). The work aims to identify unique genes that are differentially expressed as specific markers of bone marrow-derived MSCs, and to achieve this undertakes a detailed analysis of three independent datasets that include quantification of the global gene expression profiles of three primary cell types: HSCs, MSCs and FIBs. A robust bioinformatics method, called *GlobalTest*, is used to assess the specific association between one or more genes expressed in a sample and the outcome variable, that is, the ‘cell type’ provided as a single univariate response. This outcome variable is predicted for each sample tested, based on the expression profile of the specific genes that are used as input to the test. The precision of the tests is calculated along with the statistical sensitivity and specificity for each gene in each dataset, yielding four genes that mark MSCs with high accuracy. Among these, the best performer is the protein-coding gene Transgelin (TAGLN, Gene ID: 6876) (with a Positive Predictive Value > 0.96 and FDR < 0.001), which identifies MSCs better than any of the currently used standard markers: ENG (CD105), THY1 (CD90) or NT5E (CD73). The results are validated by RT-qPCR, providing novel gene biomarkers specific for human MSCs.

## 1. Introduction

Fibroblasts (FIBs) are present in the stromal component of every organ and are the main providers of the extracellular matrix, mainly collagen, that maintains their structure [1]. Mesenchymal stem cells (MSCs), also known in some forums as Mesenchymal stromal cells, constitute a non-hematopoietic population that reside in various cellular tissues, mainly bone marrow and adipose tissue. They have fibroblastic appearance when they adhere to plastic surfaces and are able to self-renew, with multipotent capacity, and therefore, they are able to differentiate into a number of cell types, including osteoblasts, chondrocytes and adipocytes [2]. MSCs were first described by Friedenstein, who demonstrated their ability to form fibroblastic colonies (CFU-F) [3,4].

To define MSCs, the International Society for Cell Therapy (ISCT) has established several minimum criteria [5]. Currently, there is no individual biomolecular marker capable of uniquely identifying MSCs. Some authors have suggested that MSCs and fibroblasts derive from a common ancestor and share most of their features [6]. In this regard, both cell types show a great resemblance in terms of morphology, adherence to plastic and proliferation capacity [7]. In terms of surface proteins, most of the so-called fibroblast-specific markers have been found to be present on MSCs and vice versa [1], and they share many phenotypic characteristics but lack the differentiation and colony-forming potential of MSCs [8]. In addition, despite their similarities, the expression levels of markers common to these two cell types vary considerably depending on the passage of the cells and the tissue of origin [9]. In terms of differentiation, both MSCs and FIBs seem to be able to change their phenotype under certain conditions into other cell types, such as osteoblasts, myoblasts, adipocytes and chondrocytes [10,11,12,13], although there is an ongoing controversy about the real differentiation capacity of FIBs, and even between MSCs of different origin (bone marrow, adipose tissue or umbilical cord blood) [14]. It is also debatable which MSC subtypes are truly multipotent [15], as they are often considered to be simple stromal cells. Blasi and co-workers suggested that the anti-inflammatory and angiogenic activity of adipose-derived MSCs (AT-MSCs) is much higher than that of fibroblasts [7]. Differences in methylation patterns have also been well reported, with FIBs having fewer CpG sites than MSCs [16].

Whole-gene expression profiles (GEPs), derived from isolated cells, allow the identification of gene signatures specific to individual cell types [17,18]. However, many published GEPs show that MSCs and FIBs have similar expression patterns, although with some relevant differences, mainly in transmembrane genes, tumor/cancer-associated genes [19] and genes related to cellular stemness and development [20,21,22]. In addition, MSCs are by far the most widely chosen option for clinical development, with 1290 registered studies using MSCs in clinical trials (https://clinicaltrials.gov/, accessed 25 July 2024) [9,23]. Currently developed genome-wide omics technologies, and in particular full transcriptome profiling methods, are very powerful tools to achieve a detailed characterization of the genes that are expressed and activated in each different cell type. In this work, we have generated a full transcriptome profiling of human bone marrow-derived MSCs (BM-MSCs) and performed a robust analysis of three datasets that provide a complete quantification of the gene expression of three different cell types: HSCs (hematopoietic stem cells), MSCs and FIBs. Each data set is independent and has been produced with different transcriptomic platforms: gene expression microarrays (from Affymetrix, Santa Clara, CA, USA), exon expression microarrays (also from Affymetrix) and RNA-sequencing (from Illumina, San Diego, CA, USA). Using these data, and our previous studies, to characterize the transcriptomic portrait of human mesenchymal stromal/stem cells [24,25], we perform a series of analyses in order to identify specific genes that are expressed in a distinct and differential manner in human MSCs. Moreover, we use a robust bioinformatic method, called *GlobalTest*, to assess the presence of an association between one or more genes expressed in a sample (measured as a set of ordinally scaled covariates) and the outcome variable as a single univariate response (i.e., the outcome that indicates the type of sample being tested). All these analyses were performed using a series of generalized linear models within the *GlobalTest* algorithm (bioconductor.org/packages/globaltest.html, accessed on 31 January 2022) [26,27]. In our case, the outcome identifies the type of sample and therefore the “cell type” of each sample tested based on the expression profile of specific gene or genes that are used as input to the test. *GlobalTest* therefore provides a robust statistical association method to elucidate whether sets of genes are significantly associated with a variable of interest, which in this study corresponds to the cell types tested (i.e., MSCs, HSCs and FIBs). The method is based on a prediction model to detect a response variable using the expression profile of the genes as input features. The null hypothesis tested is that the expression profile of a gene, among all the genes measured, is not associated with the response variable. The method also provides a statistical evaluation of the precision in the assignment and the strength of the association of a given feature with a specific phenotype, i.e., the response variable [28].

Given the great therapeutic potential of MSCs, well established by the International Society for Cell & Gene Therapy (https://www.isctglobal.org/, last accessed on 30 June 2024) [5], and the recent characterization of the transcriptome and global expression profiles of these cells, especially in the case of bone marrow-derived cells [14,24,25], we are in a good position to undertake a thorough search for specific gene markers of these elusive and not very abundant stem cells. Furthermore, as mentioned above, there is currently no single biomolecular marker capable of uniquely identifying MSCs, so it is necessary to find such specific markers. Therefore, in this study, we perform a molecular characterization of MSCs isolated from bone marrow to find such specific biomarkers, as well as to gain a better understanding of the exact composition of these cellular products used for therapeutic purposes and to provide novel approaches to explore the potential differences between similar cell types.

## 2. Results

### 2.1. Compendium of Genes Expressed in MSC, HSC and FIB

Since the main objective of our study was to perform a deep and robust search for genes that can be specific markers of human MSCs (especially those from the bone marrow), we performed a supervised search for a compendium of candidate genes that are markers of these stem cells reported in the literature, together with other genes expressed in cell types related to the MSCs: (i) the hematopoietic stem cells living in the same microenvironment of the BM; (ii) other stromal or mesenchymal cells that are not stem cells, such as fibroblasts (very abundant in many parts of our body); (iii) other MSCs isolated from different parts of the body (such as adipose tissue or placenta); and finally, a set of housekeeping and stemness genes that can be used as positive controls. As a negative control, we also included a set of genes that are not expressed in any of the cell types described above, but are characteristic of differentiated cells: adipocytes, osteocytes and chondrocytes. This is used as a negative reference set. Following this strategy, we built a compendium of 156 genes associated as cell markers with all these different cell types described above. This compendium was derived from our previous studies on the transcriptomic profiling of human MSCs and HSCs [22,24,25], but also from the literature and our current knowledge. The list of these 156 genes, with details on the main cell type in which they are expressed and active, is presented in Supplementary Table S1.

**Figure 1:**
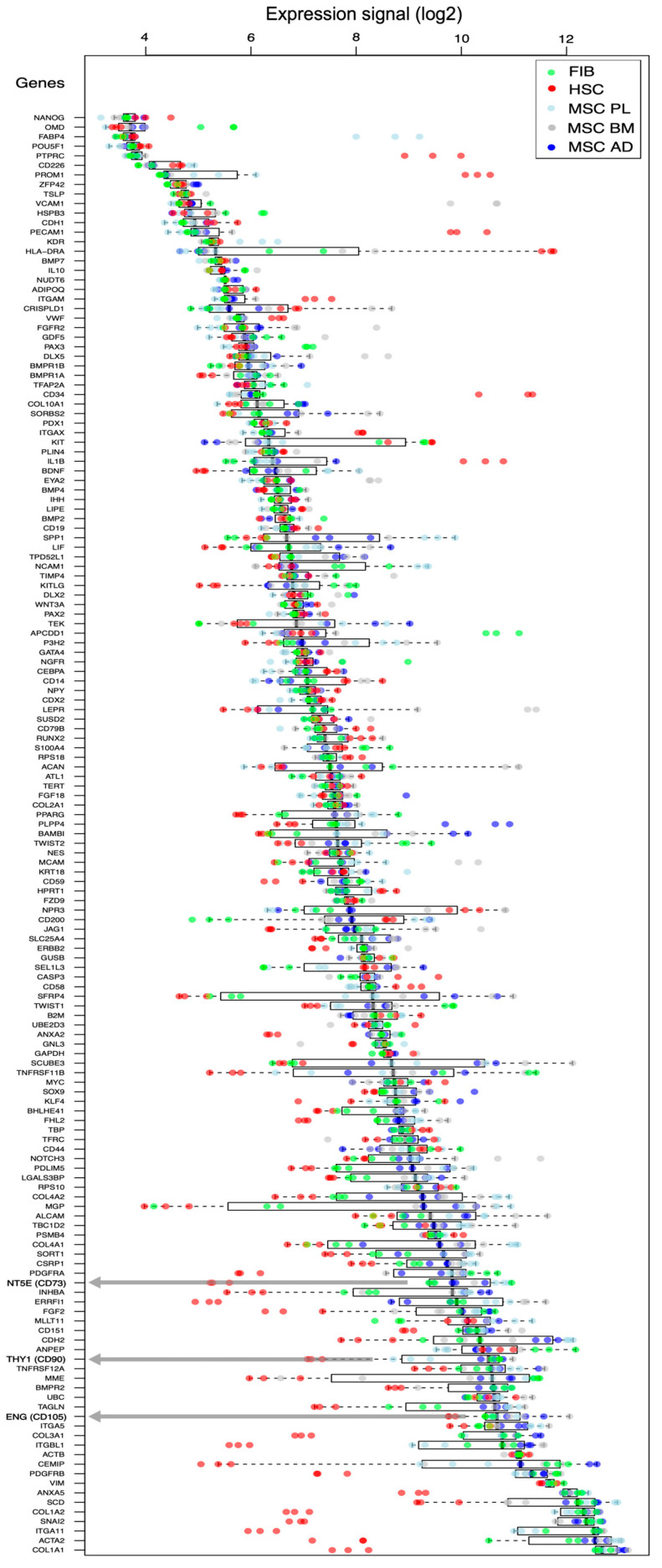
Expression signal of 151 genes selected as markers of MSCs or related cell types (the complete gene set is listed in Supplementary Table S2). The graph represents the normalized expression signal in exon arrays of these genes in 15 samples (3 samples of AD-MSCs, 3 BM-MSCs, 3 PL-MSCs, 3 HSCs and 3 FIBs), showing 151 boxplots and marking each of the samples with colored dots: FIBs (green), HSCs (red), AD-MSCs (dark blue), BM-MSCs (grey), PL-MSCs (light blue). The boxplots for the expression of each gene show the central 50% of the data as white rectangular boxes (i.e., the IQR 25-75% interquartile range), and the dashed lines show the upper quartile (75–100%) and the lower quartile (0-25%). The 3 genes that are currently used as standard markers for MSCs, ENG (CD105), NT5E (CD73) and THY1 (CD90), are marked with grey arrows.

After selecting this compendium of 156 gene markers, we search for the presence of these genes in the expression signal of the three platforms corresponding to the three independent gene expression datasets that we collected and prepared for this study (described in the Materials and Methods section). This ID mapping of the 156 genes in the three transcriptomic platforms is presented in Supplementary Table S2, which shows that dataset 1 loses 16 genes (not present in the gene microarray platform), dataset 2 loses 5 genes (not present in the exon array platform) and dataset 3 also loses 16 genes (not present in the RNA-seq platform). The genes missing in each platform are listed in Supplementary Table S2. Since the platform containing the most genes (151 in total) is the exon-array platform, we used this dataset to perform an exploratory analysis of the expression signal of these genes, and thus we present in Figure 1 the normalized expression signals of these 151 genes, showing 151 boxplots and marking each of the 15 samples included in the plots with colored dots.

In Figure 1, we have marked with an arrow the three genes that are currently used as standard markers for MSCs: ENG (CD105), NT5E (CD73), THY1 (CD90). The expression pattern of these three genes shows that they are highly expressed in the samples tested (since the genes are arranged according to the increasing median expression of the boxplots), and also shows that the MSC samples (nine blue and grey dots) are quite well separated from the HSC samples (three red dots), but not very well from the fibroblast samples (three green dots). A detailed observation of the comparative expression patterns of these genes, presented by the boxplots (Figure 1), hints that some genes that show a good separation between the MSC samples and the HSC samples, but also between the MSCs and the FIBs.

### 2.2. GlobalTest to Identify the Best Markers for MSCs in Three Independent Datasets

**Figure 2:**
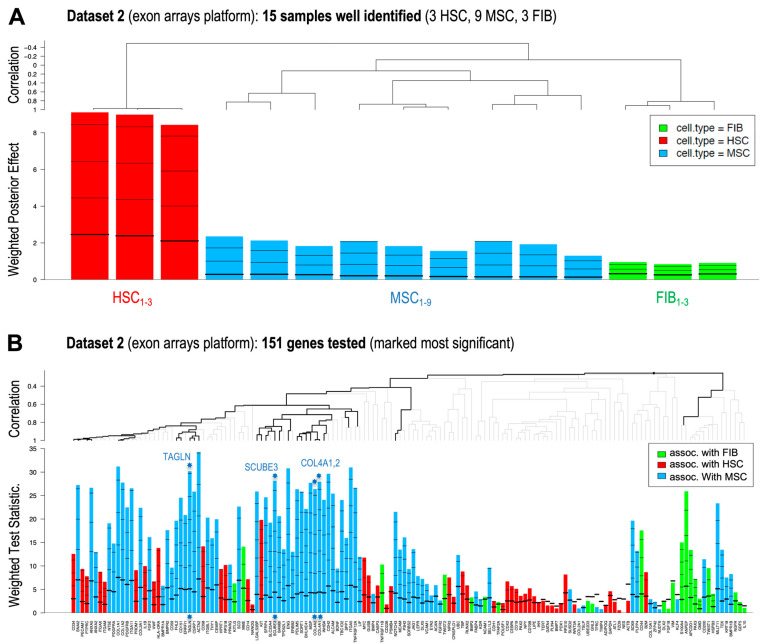
Plots showing the results of the *GlobalTest* analysis performed on expression Dataset 2, which includes 15 samples corresponding to 3 different cell types: 3 HSCs, 9 MSCs and 3 FIBs. The analysis was performed using 151 genes as selective features (gene list shown in Supplementary Table S2). Panel (**A**) shows a plot with the output of *GlobalTest* on the samples showing the confidence of their assignment to one of the 3 cell types (this confidence is measured as the weighted posterior effect). Above this plot, there is a dendrogram showing the correlation between the samples and the inferred clustering of these samples. Panel (**B**) presents a plot of the weighted test statistic for each gene (i.e., for 151 genes tested) and the cell type to which the gene is assigned or associated as a marker: FIB, green (3 samples from FIB_1_ to FIB_3_); HSC, red (3 samples from HSC_1_ to HSC_3_); MSC, blue (9 samples from MSC_1_ to MSC_9_). The blue heptagonal stars mark the position of the 4 selected genes that show a significant correlation and some of the highest weighted statistics of the GlobalTest. All these genes mark MSCs (blue bars).

In order to obtain a statistically weighted identification of the best feature variables (genes) that can be used as markers for the identification of the specific cell types included in our study, focusing on MSCs, we performed a *GlobalTest* with the three independent datasets of 70, 15 and 29 samples generated by three transcriptomic platforms. Thus, we tested whether our gene list allowed a correct and significant identification of each sample with its corresponding cell type: MSCs, FIBs or HSCs. The results are shown in Figure 2.

Figure 2 shows the results of the *GlobalTest* for the dataset of 15 samples (Dataset 2) analyzed using the exon array expression platform. The same analyses were performed for the dataset of 70 microarray samples (Dataset 1) shown in Supplementary Figure S1 and for the dataset of 29 RNA-seq samples (Dataset 3) shown in Supplementary Figure S2. The figure in panel A (i.e., Figure 2A) presents a plot with the output of *GlobalTest* on the samples showing the confidence of their assignment to one of the three cell types (this confidence is measured as the weighted posterior effect). Above this plot, there is a dendrogram showing the correlation between the samples and the inferred clustering of these samples. In summary, all of these parameters show that using this large list of candidate gene markers (i.e., the list of 151 genes with Dataset 1; 140 genes with Dataset 2; and a further 140 genes with Dataset 3), we obtain a perfect assignment of class (i.e., of cell type) to each one of the 114 samples tested. In addition, the *GlobalTest* algorithm also allows us to measure the relative weight and significance of each gene, as shown in plot B, which presents the weighted test statistic for each gene as well as the cell type to which the gene is assigned as a marker (see Figure 2B).

After this *GlobalTest* analysis, we examined the statistical parameters obtained for the three datasets, selecting the covariates (genes) that gave the best results for the identification of MSCs: TAGLN, COL4A2, COL4A1, SCUBE3 (Table 1). In addition, we also examined the same parameters for the standard MSC gene markers, namely NT5E, THY1 and ENG, and for some well-known hematopoietic gene markers used to identify HSCs, namely CD34 and CD45 (PTPRC) (Table 1). Thus, this preliminary *GlobalTest* analysis, performed using the list of 140–151 candidate gene markers, allows the identification of a reduced number of genes that should be further tested.

**Figure 3:**
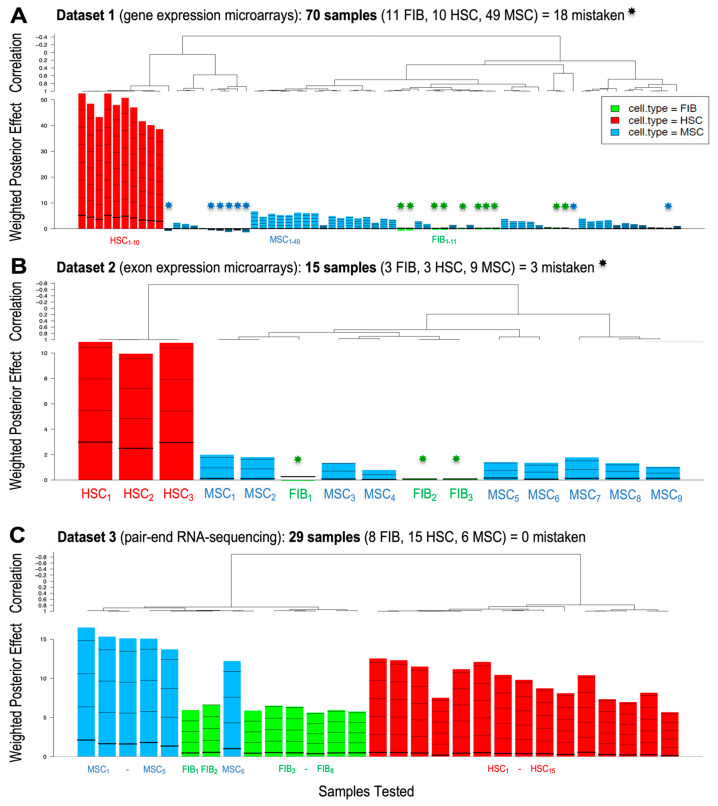
Plots of the *GlobalTest* analyses performed on 3 different expression datasets testing three cell types (HSCs, MSCs and FIBs) and using the MSC standard genes: ENG (CD105), THY1 (CD90) and NT5E (CD73). (**A**) Weighted posterior effect plot and correlation plot showing 8 misclassified samples (i.e., samples not well classified marked with asterisks) in Dataset 1. (**B**) Weighted posterior effect plot and correlation plot showing 3 misclassified samples in Dataset 2. (**C**) Weighted posterior effect plot and correlation plot for Dataset 3. In this case, all samples were well classified. The heptagonal stars (in **A**,**B**) mark the misclassified samples. The number of samples corresponding to each cell type is indicated by a subscript number.

Once these genes were pre-selected, a second set of *GlobalTests* was performed using the standard MSC gene markers as candidate markers: ENG (CD105), THY1 (CD90) and NT5E (CD73). The results of this analysis, presented in Figure 3, show the output of the *GlobalTest*, revealing how many of the 114 samples were well identified and classified into the correct cell type. In the case of Dataset 1 (Figure 3A), we found 18 incorrect samples (i.e., samples that were not well classified). In the case of Dataset 2 (Figure 3B), we found three incorrect samples that were not well classified. Finally, in the case of Dataset 3 (Figure 3C), all samples were well classified. All the misclassifications observed correspond to MSC samples being classified as FIBs or vice versa.

**Figure 4:**
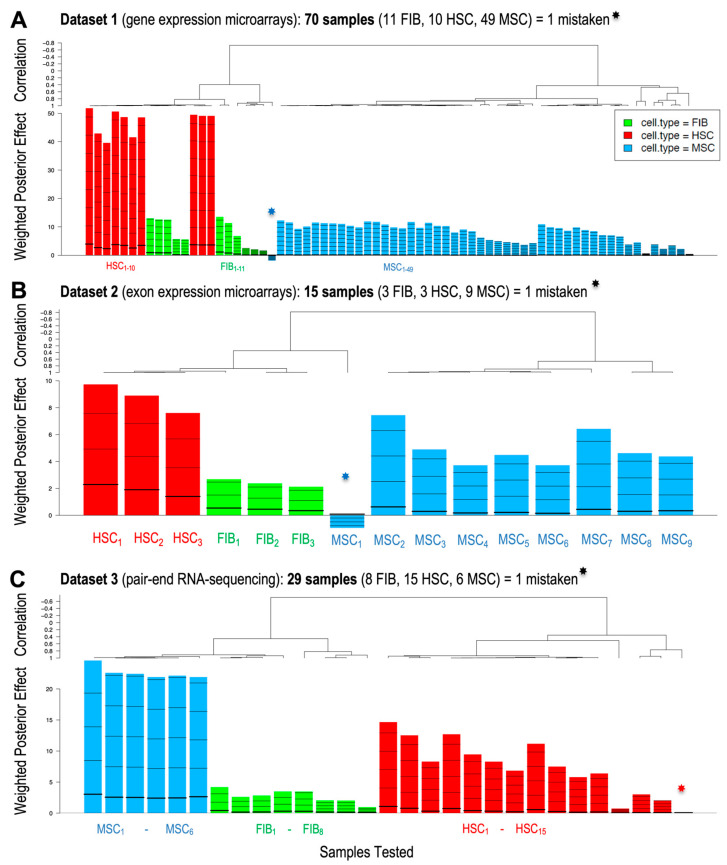
Plots of the *GlobalTest* analyses performed on three different expression datasets testing three cell types (HSCs, MSCs and FIBs) and using the genes TAGLN, COL4A1, COL4A2 and SCUBE3. (**A**) Weighted posterior effect plot and correlation plot showing only one misclassified sample in Dataset 1 (marked with an asterisk). (**B**) Weighted posterior effect plot and correlation plot showing one misclassified sample in Dataset 2. (**C**) Weighted posterior effect plot and correlation plot for Dataset 3 showing also one misclassified sample. The heptagonal stars (one in **A**, one in **B**, and one in **C**) mark the misclassified samples. The number of samples corresponding to each cell type is indicated by a subscript number.

*GlobalTests* were performed in the same way, now using the four preselected novel genes as candidate MSC markers: TAGLN, COL4A2, COL4A1, SCUBE3. The results of this analysis are in Figure 4, which shows how many of the 114 samples were well classified into the correct cell type. In this analysis, only three samples were incorrect: one MSC in Dataset 1 (Figure 4A); one MSC in Dataset 2 (Figure 4B); and one HSC in Dataset 3 (Figure 4C, which is just on the border of significance for a correct identification as HSC). All statistical parameters calculated with the *GlobatTest* for each one of the 114 samples using either the three standard MSC gene markers (ENG, THY1, NT5E) or the four novel MSC markers (TAGLN, COL4A2, COL4A1, SCUBE3) are shown in Supplementary Table S3, where the correct assignment of each sample to the corresponding the class (FIBs, class 1; HSCs, class 2; MSCs, class 3) is given when the *p*-value of the outcome assay is <0.05.

### 2.3. Statistical Parameters Derived from the GlobalTest to Identify MSCs

**Figure 5:**
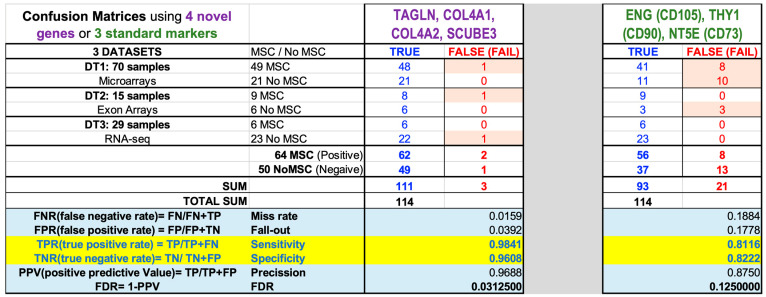
Statistical parameters derived from the *GlobalTests* used to identify MSCs. The results are presented in an illustrated table that includes the confusion matrices to show the statistical measurement of the error in the assignment to MSC cell type of samples from 3 independent datasets (DT1, DT2 and DT3, including a total of 114 samples produced with 3 different expression platforms), tested using as multivariate factors the 3 genes that are standard markers of MSCs—ENG (CD105), THY1 (CD90) and NT5E (CD73)—or using 4 novel genes selected in this study as possible new markers of MSCs—TAGLN, COL4A1, COL4A2 and SCUBE3. The statistical parameters calculated are Sensitivity and Specificity (both with values from 0 to 1), Precision (also with values from 0 to 1), FDR (*p*-value), Miss-rate and Fall-out.

With all these numbers, since we know *a priori* the cell type of each sample, we can construct a confusion matrix and calculate all the corresponding statistical parameters derived from the errors (i.e., the samples of a known cell type that were misclassified): Sensitivity (defined as True Positive Rate (TPR) = TP/[TP + FN]), Specificity (defined as True Negative Rate (TNR) = TN/[TN + FP]), Precision (defined as Positive Predictive Value (PPV) = TP/[TP + FP]), Miss-rate (defined as False Negative Rate (FNR) = FN/[FN + TP]), Fall-out (defined as False Positive Rate (FPR) = FP/[FP + TN]) and FDR *p*-value (defined as 1-PPV). All these statistical parameters calculated for the two groups of genes tested above are shown in Figure 5, which presents a colored illustrated table that divides the samples of each dataset into MSC or No MSC subtypes and indicates how many samples are truly or falsely assigned to these categories. As indicated, these results correspond to the use of three genes (ENG, THY1, NT5E—standard MSC markers) or four genes (TAGLN, COL4A2, COL4A1, SCUBE3—candidate novel MSC markers).

**Figure 6:**
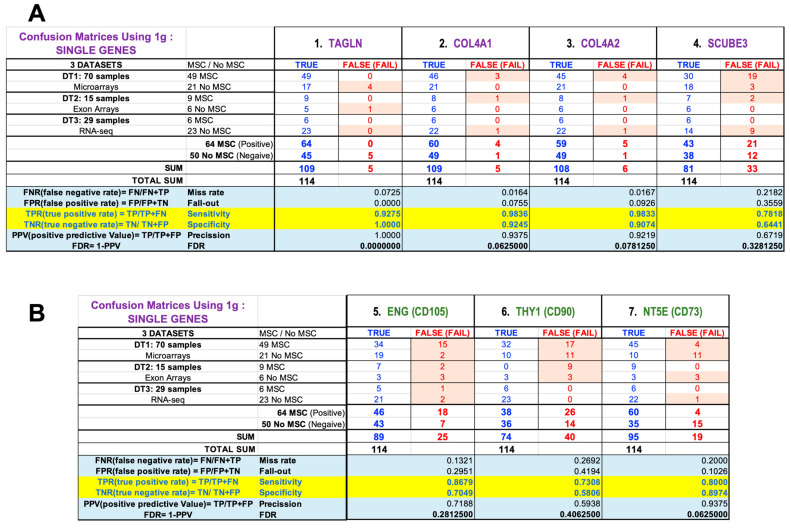
Statistical parameters derived from the *GlobalTests* used to identify MSCs. The results are presented in an illustrated table that includes the confusion matrices to show the statistical measurement of the error in the assignment to MSC cell type, of samples from 3 independent datasets (DT1, DT2 and DT3, with a total of 114 samples produced with 3 expression platforms), tested with each individual gene separated as a univariate factor: (**A**) genes TAGLN, COL4A1, COL4A2 and SCUBE3; (**B**) genes ENG (CD105), THY1 (CD90) and NT5E (CD73). The statistical parameters calculated for each gene are Sensitivity and Specificity (both with values from 0 to 1), Precision (also with values from 0 to 1), FDR (*p*-value), Miss-rate and Fall-out.

In the previous analyses, the statistical parameters derived from the *GlobalTest* results were calculated considering multiple features (i.e., using multiple markers together in a multivariate study), corresponding to the two groups of genes: the three standard markers (ENG, THY1, NT5E) and the four novel candidate markers (TAGLN, COL4A2, COL4A1, SCUBE3). Furthermore, the results corresponding to each individual gene were calculated to evaluate the discriminatory power or specificity of each single gene (with the results presented in Figure 6). From these numbers, we clearly observed that TAGLN (transgelin, gene ID: 6876) is the best gene marker of all those tested, presenting the best overall precision (value = 1.0), very good sensitivity (=0.927) and specificity (=1.0) and an FDR equal to 0. No single gene of all those tested gave such good results, because COL4A1 showed the second best results (also presenting only five errors or misclassifications), but a precision value of 0.9375 (and an FDR = 0.0625) due to the fact that four of the errors were false positives (that is, four samples that were not MSCs were classified as MSCs). The third novel gene that behaves best is COL4A2, with a precision value of 0.9219 (and an FDR = 0.0781). SCUBE3 is a gene that does not perform well because it shows many misclassifications in the confusion matrix (Figure 6A). Finally, in all cases, the TAGLN gene shows fewer errors in the identification of MSCs than any of the three standard genes (CD105, CD90 and CD73), which often showed clear difficulties in the correct identification of FIBs versus MSCs (Figure 6B).

### 2.4. Validation of the Expression Signal of the Genes by qPCR in Different Cell Types

**Figure 7:**
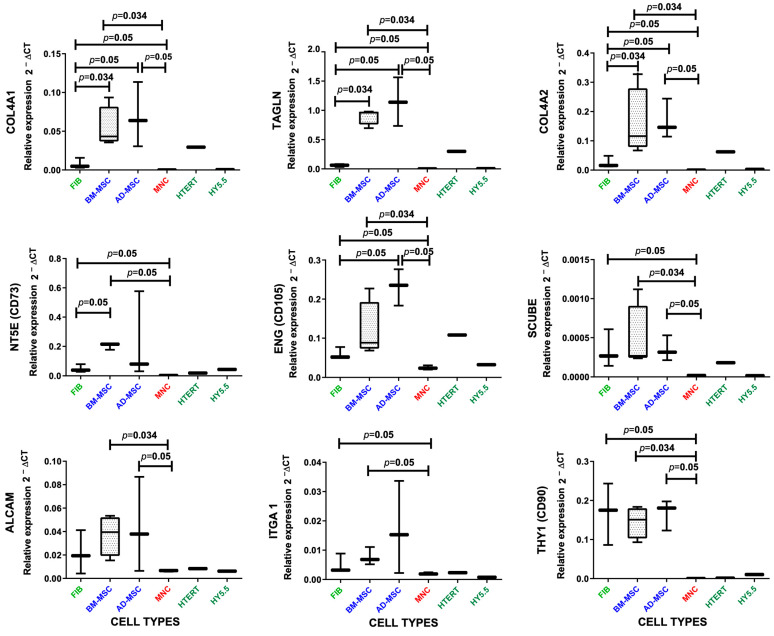
Validation of the expression signal of the genes using RT-qPCR. Nine graphs corresponding to 9 tested genes (COL4A1, TAGLN, COL4A2, NT5E, ENG, SCUBE, ALCAM, ITGA1 and THY1), each including 6 box plots showing the expression level of the corresponding gene in 6 different cell types: fibroblasts (FIBs); MSCs from two different sources (BM-MSCs and AD-MSCs); mononuclear cells (MNCs) isolated from bone marrow; and two human stromal cell lines (HTERT and HY5.5). The *p*-values for pairwise comparisons of the box plots are included in each graph when significant (≤0.05).

All these results were validated in an independent way using RT-qPCR with new samples from six human cell types. The results are shown in Figure 7, which confirms the expression level of nine genes measured in samples from six different cell types: MSCs from two different sources (BM-MSCs and AD-MSCs); fibroblasts (FIBs); mononuclear cells (MNCs) isolated from bone marrow; and two human stromal cell lines (HTERT and HY5.5). The results indicated again that COL4A1, COL4A2 and TAGLN are good and distinctive markers of MSCs from bone marrow or adipose tissue, highly expressed in these cells and with very low expression in the other cells tested. In fact, these genes showed very significant differences between any type of MSC versus fibroblasts (Figure 7). When comparing fibroblasts with MSCs, the expression of NT5E (CD73) was also significantly higher in MSCs from bone marrow (BM-MSCs) but not in MSCs from adipose tissue (AD-MSCs). In fact, gene marker CD73 was the one that gave the best results in the statistical analysis performed with the *GlobalTests*, with an overall precision of 0.9375 (Figure 6B). In contrast, the other two standard MSC markers CD105 (ENG) and CD90 (THY1) did not show significant differences between BM-MSCs and fibroblasts (see the box plots for genes ENG and THY1 in Figure 7). Regarding the comparison between fibroblasts and MSCs from adipose tissue (AD-MSCs), besides TAGLN, COL4A1 and COL4A2, the gene endoglin (ENG, gene ID: 2022) is the only standard MSC marker that shows a significant difference with the FIBs (Figure 7), displaying a higher expression in the boxplots.

In conclusion, our results confirm that the genes TAGLN, COL4A1 and COL4A2 could be used as specific markers of human mesenchymal stem cells (MSCs), showing clear differences with fibroblasts (FIBs). Out of these three genes, the one that provides best results in a consistent way is TAGLN (transgelin; Entrez Gene ID: 6876, Ensembl ID: ENSG00000149591, HGNC ID: 11553).

## 3. Discussion

Despite their identification many years ago, fibroblasts remain largely unknown in relation to other mesenchymal cells, perhaps due to their presence in most organs and their heterogeneity. Currently, researchers are still trying to find markers that can distinguish MSCs from FIBs, and the exact relationship between these two stromal cell types has long remained misunderstood [29]. In fact, the elusive nature and function of mesenchymal stem cells has been reported in well-documented reviews for more than a decade, such as the one by Nombela-Arrieta et al. in 2011 [30], the review by Uccelli et al. in 2008 [31] or the more recent study by Mabuchi et al. in 2021 [32] that focused on the cellular heterogeneity of MSCs in bone marrow.

The development of reproducible genome-wide omics techniques, together with the generation of complete transcriptomic profiles for many specific cell types and subtypes, has opened a new avenue to better differentiate cell types that are very similar, such as MSCs and FIBs [1]. Indeed, these cells share many aspects such as surface markers [1], immunomodulatory properties, methylation patterns [16] and differentiation and proliferation capacity [7]. The results presented in this work have identified specific gene markers for human MSCs found in robust comparison with other related cell types. In particular, the search for gene markers that allow a clear distinction between MSCs and FIBs is a relevant and novel objective because, nowadays, the canonical standard CD markers (i.e., the genes ENG (CD105); THY1 (CD90); and NT5E (CD73)) used to identify and select human MSCs because they are highly expressed in these cells are also expressed at similar levels in FIBs [25,33]. Therefore, MSCs can often be mistaken for FIBs and vice versa. To address this issue, we perform deep analyses of multiple genome-wide expression data to find novel genes that show more distinct expression in MSCs versus other cell types.

NT5E (CD73) catalyzes the conversion of extracellular nucleotides into membrane-permeable nucleotides. This marker is present in a wide variety of cells, and in this study (Figure 6B), we showed that NT5E is the standard MSC marker that shows the greatest difference with FIBs (in the case of BM-MSCs). Consistent with these results, Luo et al. showed that NT5E and ALCAM expression levels were higher in MSCs than in FIBs [33]. THY1 (CD90) has been found in many cells and is involved in cell–cell and extracellular matrix interactions [34]. However, CD90 as a cell marker performed poorly both in the identification of MSCs in the *GlobalTest* assays (Figure 6B) and in the experimental comparisons by qPCR (box plot in Figure 7). In fact, using THY1 (CD90), we found no statistically significant differences between the three cell types: BM-MSCs, AD-MSCs and FIBs. Finally, ENG (CD105 marker) showed a clearer expression difference between MSCs and FIBs. This gene has been shown to increase in expression with increasing passage rates in the in vitro MSC cultures [35]. Taken together, our results indicate that none of these three standard MSC markers (CD73, CD90, CD105), which must be positive in multipotent mesenchymal stem cells (according to the International Society for Cell & Gene Therapy, ISCT www.isctglobal.org, last accessed on 30 June 2024) [5], would be valid as a fully specific marker for these cells, especially in distinguishing them from FIBs.

After analyzing the transcriptomic profiles of several cells related to MSCs (related either by the stromal lineage or by the stemness lineage), we found four candidate protein-coding genes: TAGLN, COL4A1, COL4A2 and SCUBE3. TAGLN (transgelin, gene ID: 6876) belongs to the calponin family and is involved in calcium-independent muscle contraction, actin binding and cytoskeletal organization. COL4A1 and COL4A2 (gene IDs: 1282 and 1284) encode type IV collagen alpha proteins and are involved in the formation of the extracellular matrix (type IV collagen proteins are integral components of basement membranes). SCUBE3 (protein-coding gene for Signal peptide, CUB and EGF-like domain-containing protein 3, gene ID: 222663) is a gene encoding a secretory cell surface glycoprotein that plays a key role in the developmental process and promotes epithelial–mesenchymal transition. Note that of these four candidate markers, TAGLN was the gene that gave the best and most consistent results with the fewest errors in identifying the cell types tested (Figure 6A); however, SCUBE3 showed many errors in identifying the cells, with an overall accuracy of only 67% (i.e., a positive predictive value of 0.67). Therefore, SCUBE3 is ultimately not recommended as a suitable marker for MSCs.

It has been reported that the expression profile of the same cell types can vary depending on their source in the body (i.e., the tissue or organ of origin). For example, the expression profiles of different genes in MSCs isolated from adipose tissue are not the same as in MSCs isolated from bone marrow [21]. We see these differences in our results for different genes.

Previous studies have shown overexpression of TAGLN after addition of TFGB1 (transforming growth factor beta 1) in mesenchymal stem cells derived from the hTERT cell line and in other cell types such as smooth muscle cells. This treatment increased the ability of these cells to differentiate into both osteoblastic and adipogenic lines, as well as their motility and migration, but decreased their proliferation. Meanwhile, gene silencing had the opposite effect in the same cells [36]. As a gene involved in the organization of the cytoskeleton, it is believed that during the differentiation, MSCs change their morphology and arrangement of actin filaments due to overexpression of TAGLN, along with other molecules also involved: NRIH3, CDF, IGFBP2 and IGF2. In line with our results, Elsafadi et al. used skin fibroblasts as a control in their studies on MSCs. They observed via Western blotting that the FIBs did not contain the TAGLN protein, whereas it is present in the MSCs from the hTERT cell line (although their study is not based on a direct comparison between the two cell types) [36]. Brun and coworkers observed the same in MSCs from three healthy donors in the presence or absence of myogenic differentiation medium under GMP conditions [37]. Therefore, TAGLN has been frequently used as a marker of smooth muscle differentiation. In addition, the study of TAGLN expression levels in different cells from human bladder tissue showed that FIBs have lower expression than endothelial and smooth muscle cells [38]. The low expression of TAGLN in healthy fibroblasts has also allowed its use as a pathological marker. Indeed, it has recently been used as a biomarker for poor prognosis in patients with rectal colon cancer due to its strong overexpression in FIBs associated with primary tumor cells [39]. The same is true in skin fibroblasts from patients diagnosed with systemic sclerosis [40] and when they compared TAGLN expression between control and UV-irradiated skin fibroblasts [41]. This higher expression of TAGLN observed in tumor-associated FIBs suggests that the gene may be related to cell immortalization and stemness.

Regarding the variability of TAGLN expression between MSCs derived from different tissues, this correlates with the intrinsic heterogeneity of MSCs of different tissue origins (such as bone marrow, adipose tissue, cord blood, etc.) [30], as is clearly reflected in the last figure presented here (Figure 7), where we show the expression of nine genes in two types of MSCs: BM-MSCs and AD-MSCs. Despite these observed changes, the expression level of TAGLN in the different MSCs is more similar than its expression in cell types of different cellular lineages (such as FIBs and MNCs) (Figure 7).

Muhl and coworkers characterized FIBs from four muscle organs (head, squeletic muscle, bladder, and colon) of mice and compared them with pericytes using single-cell techniques. The list of genes detected in this single-cell expression profiling of FIBs did not include the TAGLN gene. However, TAGLN was a pericyte or vascular mural cell marker in many of the mouse organs analyzed [42]. Regarding the other novel gene markers found in our results, COL4A1 and COL4A2, there are no references in this study [42]. COL4A1 and COL4A2, along with many other COL genes, are involved in the synthesis of collagen, the main structural protein in the extracellular matrix of the human body. It has been shown that COL4A1 expression in skin FIBs decreases progressively with age [43]. Another recent study compared the genome-wide expression profiles of three types of skin FIBs (isolated from the dermo-hypodermal junction, from the intermediate reticular dermis and from the superficial papillary dermis) with MSCs from different sources and found that COL4A2 expression was higher in the case of dermo-hypodermal junction FIBs compared to MSCs from adipose tissue [44]. This particular observation does not agree with our results, which reflect a significantly higher expression of COL4A1 and COL4A2 in BM-MSCs and AD-MSCs compared to skin FIBs, but it also shows that FIBs from different sources can have quite different expression levels for the collagen structural genes, as they are a very complex superfamily of genes. Finally, SCUBE3 is a glycoprotein capable of enhancing growth factor signaling. This receptor is highly expressed in osteoblasts and chondrocytes. It maintains a close relationship with bone morphogenetic proteins (BMPs) by facilitating and stabilizing BMP receptors [45]. These properties may justify its higher expression in the MSCs from our study.

In conclusion, our results using genome-wide expression profiling identify four genes capable of distinguishing between MSCs of two different origins (adipose tissue and bone marrow) and skin FIBs. However, the best new marker found for human MSCs is TAGLN, since it shows the best and most robust specificity, with clear potential to replace the existing markers. In fact, the results presented here, produced with multiple comparisons, demonstrate that TAGLN outperforms and surpasses in expression signal the ability to distinguish MSCs from FIBs and HSCs with respect to the current widely used standard positive markers for MSCs: CD73, CD90 and CD105.

In this study, we have only worked at the level of gene expression and therefore have not performed experiments to test the identified gene markers at the protein level (e.g., using specific antibodies to detect the proteins). However, a search in the Human Protein Atlas database (HPA, https://www.proteinatlas.org/, last accessed on 30 June 2024) showed that the proteins corresponding to the gene markers proposed here have similar expression at both the transcript and protein levels.

## 4. Materials and Methods

### 4.1. Datasets Compiled and Used to Test for Novel Gene Markers

Three independent datasets including genome-wide expression data from three different human primary cells: hematopoietic stem cells (HSC), bone marrow-derived Mesenchymal Stem Cells (MSC), and primary Fibroblasts (FIB) were collected, normalized from raw data and quantified to produce full gene expression data. The datasets were produced using three different transcriptomic platforms: (i) gene expression Affymetrix high-density microarrays (GeneChip Human Genome U133 Plus 2.0); (ii) exon expression Affymetrix high-density microarrays (Human Exon 1.0 ST Array); and (iii) Illumina pair-end RNA-sequencing technique. The first dataset included 70 samples corresponding to gene expression microarrays obtained as a selection from a set of 264 samples and 18 datasets included in a large meta-analysis of public data developed for our previous work, published in Sanchez-Luis et al. [22], where all details about the construction of this large set are provided. Therefore, this first dataset included samples of MSCs from 49 donors, isolated from bone marrow; 10 samples of HSCs from bone marrow; and 11 samples of primary FIBs isolated from skin. All 70 samples were originally taken from the public database GEO (www.ncbi.nlm.nih.gov/geo/, last accessed on 30 June 2024) and are described in [22]. The second dataset included 15 samples of exon expression arrays generated in our laboratory [25], including 3 samples of adipose-derived MSCs, AD-MSCs; 3 samples of bone marrow MSCs, BM-MSCs; 3 samples of placental MSCs, PL-MSCs; 3 progenitor hematological stem cells, HSCs; and 3 samples of primary dermal fibroblasts, FIBs. All samples were tested using the Human Exon 1.0 array platform (Affymetrix) with the probes mapped to ENSEMBL genes using the GATEexplorer algorithm [46]. This dataset is fully accessible in the GEO database under the identifier GSE72332. Finally, the third dataset corresponds to a compendium subset from 8 expression datasets, all produced with Illumina RNA-sequencing technology (Illumina HiSeq 2000, 2500 or 4000 platforms), from which we selected the raw files and normalized together 29 samples: 6 samples of BM-MSCs; 15 samples of bone marrow HSCs; and 8 samples of primary skin FIBs. The original datasets containing these samples are accessible in GEO under the following identifiers: GSE51518, GSE63569, GSE74053, GSE81478, GSE102881, GSE105145, GSE114922, GSE119501.

### 4.2. Computational Data Analysis to Identify Cell-Specific Gene Markers

Using the R computer language for statistical programming (version 4.0.4, cran.r-project.org, last accessed on 31 January 2024) and various software packages from Bioconductor (www.bioconductor.org, last accessed on 31 January 2024), we implemented a series of bioinformatic computer protocols and methods to analyze the gene expression signatures of samples from different closely related cell types (i.e., MSCs, HSCs and FIBs), with the aim of identifying one or more genes that are specific and selective markers of mesenchymal stem cells isolated from human bone marrow: BM-MSCs. As mentioned above, we have 3 independent gene expression datasets of 70, 15 and 29 samples generated by 3 different transcriptomic platforms. Using these data, we applied a robust algorithm called *GlobalTest*, which allows us to test groups of features for significant association with a response variable, i.e., to test all features (i.e., the genes) that are candidate markers associated with a given subset or type of samples (i.e., with specific cell types) [26]. The algorithm is based on multiple regression to fit the model by selecting the best features in the role of candidate markers that influence a response variable because they show a significant association or correlation with a particular class of samples [26,27]. The results provide a statistical test with adjusted *p*-value to weigh the association between the selected features (i.e., selected genes) and the output state (i.e., sample subtype or cell type) [28]. Note that the *GlobalTest* is blind to the cell type labels and has no a priori knowledge of the cell type of the samples tested, it simply reads the expression profile of each sample and based on this assigns the sample to one of the queried categories.

### 4.3. Human Cell Samples to Validate the Expression of the Markers by RT-qPCR

To confirm and validate the findings of the bioinformatic analysis conducted in the three datasets in the current study, samples from different human cell types were obtained from various sources, as described in Table 2. In this way, we obtained mononuclear cells (MNCs) from peripheral blood (n = 3 samples); mesenchymal stem cells isolated from bone marrow (BM-MSCs, n = 4 samples); and mesenchymal stem cells isolated from adipose tissue (AD-MSCs, n = 3 samples). All these were obtained from healthy donors. Furthermore, human primary fibroblasts (FIBs, n = 3) from the dermis (from 3 different female donors) were purchased from *Innoprot* (https://innoprot.com/, accessed on 31 January 2022), and two human stromal cell lines (hTERT and HS-5) were obtained as described below (SC, n = 2).

All samples from donors obtained in our hospital (MNCs, BM-MSCs and AD-MSCs) were drawn after the corresponding informed consent was obtained in accordance with the Ethical Standards and Good Clinical Practice established by the Ethics Committee of the University Hospital of Salamanca (HUS).

### 4.4. Cell Processing

Cell types were processed as follows. Isolation of mononuclear cells (MNCs) from peripheral blood: First, 5 mL of peripheral blood was collected in EDTA tubes from 3 healthy donors. Peripheral blood was lysed two times with 1% ammonium chloride for 20 min and 5 min, respectively, in the cold, and after incubation was washed twice with PBS. The pellet was mixed with trizol, and RNA was extracted. For fibroblasts (FIB), cryopreserved fibroblast cells from 3 different adults (purchased from Innoprot, Derio, Spain) were thawed and seeded at the rate of 5000 cells/cm^2^ in a culture flask with fibroblast medium supplemented with 2% Fetal Bovine Serum (FBS), 1% Fibroblast Growth Supplement (FGS) and 1% penicillin/streptomycin solution until confluence was reached. In this moment, cells were trypsinized and subcultured to obtain the needed number for further experiments. Bone marrow collection: Bone marrow extraction was performed under locoregional anesthesia by puncture in the postero-superior iliac spine. Mononuclear cells from the bone marrow were obtained by density gradient by Ficoll-PaqueTM Plus (density:1.077 k; GE Healthcare Bio-Sciences AB). Obtained cells were centrifuged for 30 min at 500 g following the method previously described by Minguell and collaborators [2]. Finally, the mononuclear cells recovered from the interphase were washed 2 times with HBSS medium (Hank’s Balanced Salt Solution with Phenol Red, BioWhittaker Lonza Verviers, Belgium) for 10 min at 300 g. Procurement of adipose tissue: Human adipose tissue was obtained from liposuction under general anesthesia. Briefly, we disintegrate 1 g of adipose tissue from the liposuction and incubate it with collagenase at 37 °C for one hour in agitation. Then, we pass the homogenized material through a 40 µm filter and centrifuge the obtained filtrate. After lysing the cell button with ACK 1% lysing buffer (A10492 Gibco, Invitrogen, Paisely, UK), mononuclear cells were washed two times with PBS [47]. Isolation and expansion of MSC from BM and AT: The mononuclear cells obtained from BM and the cells from AT were seeded at a rate of 1 × 10^6^/cm^2^ in Dulbecco’s Modified Eagle’s medium-low glucose (DMEN, Gibco Invitrogen, Paisely, UK) supplemented with 10% Fetal Bovine Serum (FBS, BioWhittaker^®^ Lonza, Verviers, Belgium) and 1% penicillin/estreptomycin (Gibco, Invitrogen, Paisely, UK). Every 2–3 days, the culture medium was changed until 80–90% confluence was reached. At this time, all culture medium was removed from the flask, washed with sterile PBS (GIBCO Invitrogen Corporation, Paisely, UK) and incubated with 0.05% trypsin (GIBCO Invitrogen Corporation, Paisely, UK) for 5 min at 37 °C. Cells were subsequently seeded at a concentration of 5000 cells/cm^2^ into culture flasks with a larger surface area, until reaching passage 3, which was used for this study [48]. Stromal cell lines: We used two human stromal immortalized cell lines (hTERT and HS-5), both grown with medium expansion of mesenchymal (DMEN, 10% FBS and 1% P/S). The first cell line, an adipose human mesenchymal stem cell line immortalized by expression of the telomerase reverse transcriptase gene (hMSC-TERT), was a generous gift from Dr. D Campana (Professor, Department of Paediatrics, Yong Loo Lin School of Medicine, National University of Singapore), whereas cell line HS-5 was derived from bone marrow stroma (HS-5, ATCC^®^ CRL-11882™, https://www.atcc.org/products/crl-3611, accessed on 31 January 2022).

### 4.5. Quantitative Gene Expression Measurements Using RT-qPCR

All cells were collected and RNA was extracted by the thiocyanate–phenol–chloroform method [49]. To measure the concentration and the RNA integrity, the Agilent 2100 Bioanalyzer system (Agilent, Palo Alto, CA, USA) was used. Reverse transcription was performed according to Van Dongen JJ [50], using the High Capacity kit (Applied Biosystems, Foster City, CA, USA). For quantitative PCR, the Step One Plus Real-Time PCR System (Applied Biosystems, Foster City, CA, USA) was used with commercial TaqMan^®^ Gene Expression Assays (Applied Biosystems, Foster City, CA, USA). The genes analyzed are shown in Table 3, along with the identification number of each gene tested. To quantify the expression of each query gene with respect to the control gene (GADPH), we calculated the formula 2-∆Ct, where ∆Ct = Ct study gene − Ct GADPH. Differences between the expression signals obtained with the different cell types were analyzed to identify the significant differences (i.e., *p*-values < 0.05) using a non-parametric Mann–Whitney U test between samples.

## Figures and Tables

**Table 1:** *GlobalTest* statistics of the selected covariates (genes) TAGLN, COL4A2, COL4A1 and SCUBE3; standard genes NT5E, THY1 and ENG; and hematopoietic genes CD34 and PTPRC (CD45). The *GlobalTest* was performed on the 151 genes in each of the three independent datasets: dataset 1 (microarrays, 70 samples), dataset 2 (exon arrays, 15 samples) and dataset 3 (RNA-seq, 29 samples). The parameters obtained for each one of the tested genes are the weighted t statistic, the mean value of the gene in all samples, the standard deviation (SD) and the number of standard deviations from the mean.

Dataset 1 (Microarrays)					
Genes	*p*-Value	Weighted t Statistic	Mean	SD	Number of SDs
TAGLN	3.66 × 10^−12^	23.1926	1.3168	1.3633	16
COL4A2	1.68 × 10^−11^	18.5605	0.9226	0.9631	18
COL4A1	5.13 × 10^−12^	20.7785	0.9849	1.0293	19
SCUBE3	1.74 × 10^−7^	9.1558	0.6176	0.645	13
NT5E (CD73)	2.18 × 10^−8^	9.7083	0.812	0.8331	10
THY1 (CD90)	9.39 × 10^−6^	8.7419	0.8639	0.8912	8
ENG (CD105)	9.45 × 10^−8^	8.106	0.5581	0.581	12
CD34	2.69 × 10^−7^	4.1783	0.3257	0.3354	11
PTPRC (CD45)	6.72 × 10^−8^	2.0112	0.2253	0.2184	8
**Dataset 2 (Exon Arrays)**					
**Genes**	***p*-Value**	**Weighted t Statistic**	**Mean**	**SD**	**Number of SDs**
TAGLN	0.0002	30.1943	5.1138	4.916	5
COL4A2	0.0003	26.395	4.2427	4.1633	5
COL4A1	0.0004	27.9514	4.4327	4.3823	5
SCUBE3	0.0003	28.1321	4.4107	4.3338	5
NT5E (CD73)	0.0017	19.0957	4.7754	4.4351	3
THY1 (CD90)	0.0265	15.9119	4.9521	4.746	2
ENG (CD105)	0.0005	30.8115	5.8109	5.47	4
CD34	0	12.5627	3.0145	2.7457	3
PTPRC (CD45)	0	7.7998	1.977	1.8009	3
**Dataset 3 (RNA-Seq)**					
**Genes**	***p*-Value**	**Weighted t Statistic**	**Mean**	**SD**	**Number of SDs**
TAGLN	4.53 × 10^−9^	31.8339	2.2771	2.2832	12
COL4A2	3.93 × 10^−9^	26.6082	1.9769	1.9697	12
COL4A1	2.26 × 10^−8^	23.4991	1.9071	1.8878	11
SCUBE3	0.0003	7.6833	1.1733	1.1453	5
NT5E (CD73)	5.11 × 10^−9^	19.8728	1.4518	1.4491	12
THY1 (CD90)	6.45 × 10^−11^	30.6339	1.8775	1.8886	15
ENG (CD105)	7.88 × 10^−6^	9.3085	1.0661	1.0393	7
CD34	2.48 × 10^−9^	26.3858	1.7843	1.7969	13
PTPRC (CD45)	1.52 × 10^−10^	29.6913	1.8414	1.8503	15

**Table 2:** Characteristics of the human samples of different cell types that were used to validate the expression of the gene markers using RT-qPCR. (The samples come from healthy donors, D1–D14, or from cell lines, L14 and L15.)

Donors/Lines	Cell Type	Cells Source	Age	Gender
D1	MNC	Peripheral Blood (PB)	18	Female
D2	MNC	Peripheral Blood	32	Male
D3	MNC	Peripheral Blood	20	Male
D4	FIB	Dermis	22	Female
D5	FIB	Dermis	38	Female
D6	FIB	Dermis	34	Female
D7	BM-MSC	Bone Marrow (BM)	24	Female
D8	BM-MSC	Bone Marrow	38	Male
D9	BM-MSC	Bone Marrow	60	Male
D10	BM-MSC	Bone Marrow	38	Male
D11	AD-MSC	Adipose Tissue (AD)	31	Female
D12	AD-MSC	Adipose Tissue	49	Female
D13	AD-MSC	Adipose Tissue	40	Female
L14 (HS-5)	SC	HS-5 cell Line	–	–
L15 (HTERT)	SC	hTERT cell line-AD	–	–

*Abbreviations*: D, Donor; L, Cell Line; MNC, Mononuclear Cells; PB, Peripheral Blood; FIB, Fibroblasts; MSC, Mesenchymal Stem Cells; BM, Bone Marrow; AD, Adipose Tissue; SC, Stromal Cells.

**Table 3:** Information about the human genes tested by RT-PCR to quantify expression in different human cells. The Catalog number and the Entrez NCBI ID are provided to check gene identity and sequence information.

Catalog No.	NCBI ID	Gene Symbol	Gene Full Name-Description
HS00977641_M1	214	ALCAM (CD166)	Activated-Leukocyte Cell Adhesion Molecule
HS00266237_M1	1282	COL4A1	Collagen Type IV Alpha 1 Chain
HS05006309_M1	1284	COL4A2	Collagen Type IV Alpha 2 Chain
HS00923996_M1	2022	ENG (CD105)	Endoglin
HS99999905_M1	2597	GAPDH	Glyceraldehyde-3-Phosphate Dehydrogenase
HS00235006_M1	3672	ITGA1 (CD49a)	Integrin subunit alpha 1
HS01573922_M1	4907	NT5E (CD73)	5′-nucleotidase ecto
HS00162558_M1	6876	TAGLN	Transgelin
HS00174816_M1	7070	THY1 (CD90)	Thy-1 Cell Surface antigen
HS00221277_M1	57758	SCUBE2	Signal Peptide, CUB and EGF Like Domain 2

## Data Availability

As indicated in the Materials and Methods section, all the datasets used in this study are available in public databases (such as Gene Expression Omnibus (GEO): https://www.ncbi.nlm.nih.gov/geo/, last accessed on 30 June 2024). Additional data supporting the results of this study are available upon request from the corresponding author (Javier De Las Rivas).

## Supplementary Materials

The following supporting information can be downloaded at https://www.mdpi.com/article/10.3390/ijms252211906/s1. These includes two supplementary Figures and three Tables: Figure S1: *GlobalTests* done expression Dataset 1 with 3 different cell-types tested using 140 genes; Figure S2: *GlobalTests* done expression Dataset 3 with 3 different cell-types tested using 140 genes; Table S1: Table including the list of 156 selected gene markers, with details of the main cell types in which they are expressed and active; Table S2: Table showing the lists of the 156 genes which are present in each of the three transcriptomic platforms studied (microarray platform, Dataset 1, detects 140 genes; exon array platform, Dataset 2, detects 151 genes; RNA-seq platform, Dataset 3, detects 140 genes); Table S3: Statistical parameters calculated with the GlobatTest for each one of the 114 samples using either the 3 standard MSC gene markers together (ENG, THY1, NT5E) or the 4 novel MSC markers together (TAGLN, COL4A2, COL4A1, SCUBE3), as well as each one of these 7 genes alone.
