# Supplementary figures and images for: Novel Gene Biomarkers Specific to Human Mesenchymal Stem Cells Isolated from Bone Marrow
Source: Int J Mol Sci. 2024 Nov 6;25(22):11906. doi: 10.3390/ijms252211906 (PMC11593895; doi:10.3390/ijms252211906)

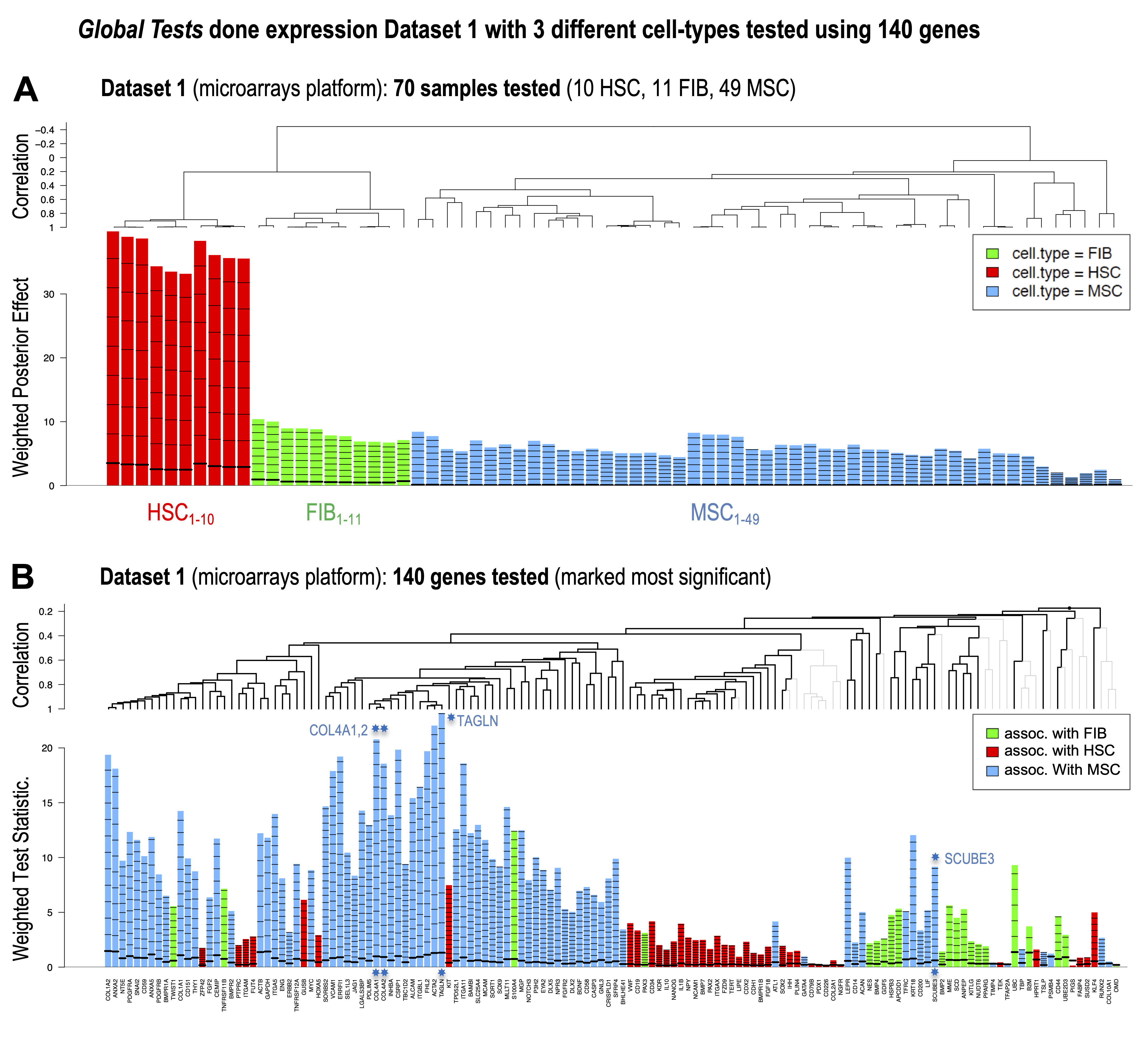

Supplement: Supplementary file 1 [file ijms-25-11906-s001.zip › ARTICLE_Muntion-et-al_Supplementary-FIGURE-S1.png]

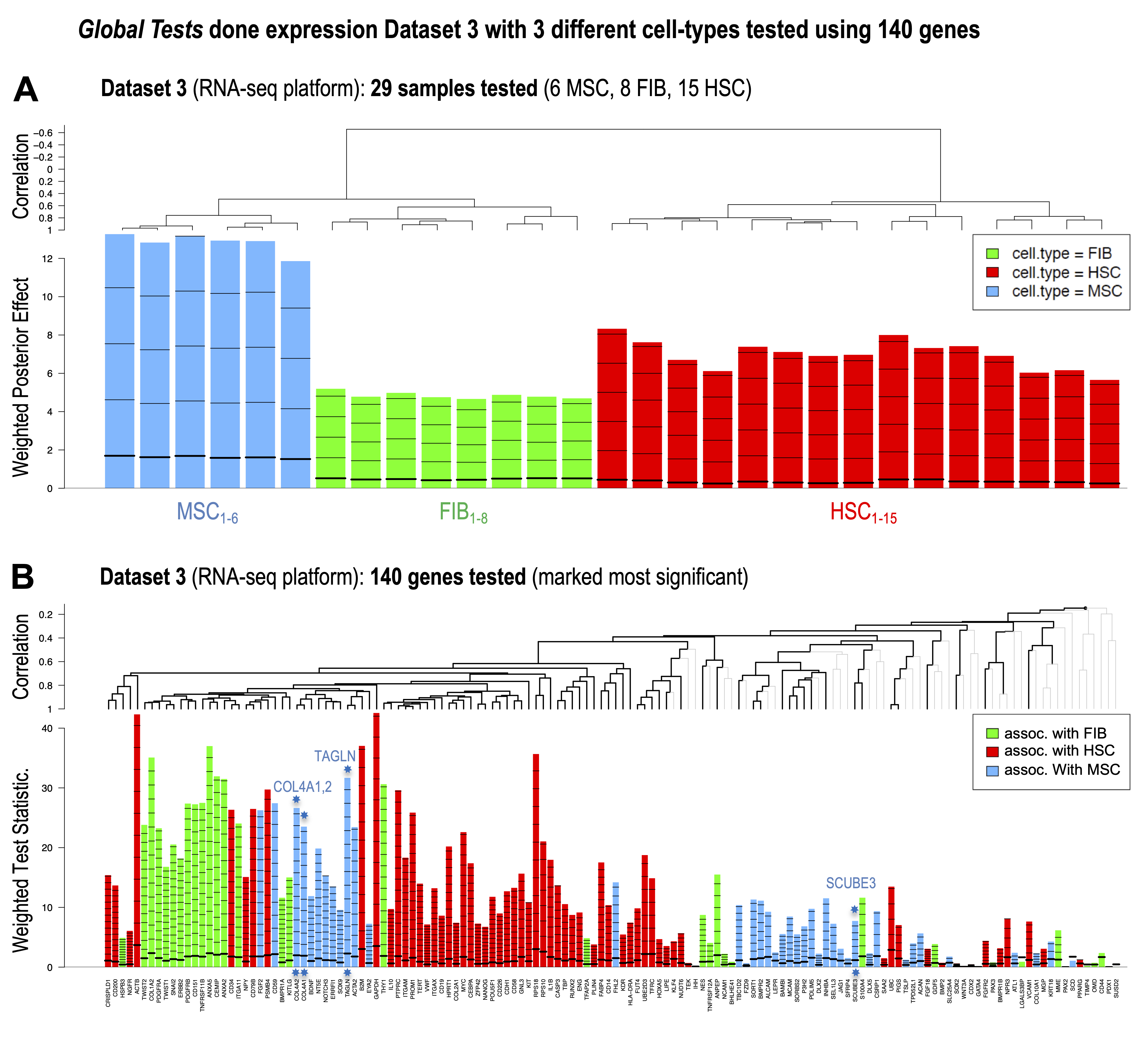

Supplement: Supplementary file 1 [file ijms-25-11906-s001.zip › ARTICLE_Muntion-et-al_Supplementary-FIGURE-S2.png]
